# *BRAF^V600E^* Mutant Allele Frequency (MAF) Influences Melanoma Clinicopathologic Characteristics

**DOI:** 10.3390/cancers13205073

**Published:** 2021-10-11

**Authors:** Xavier Soria, Felip Vilardell, Óscar Maiques, Carla Barceló, Pol Sisó, Inés de la Rosa, Ana Velasco, Dolors Cuevas, Maria Santacana, Sònia Gatius, Xavier Matías-Guiu, Alberto Rodrigo, Anna Macià, Rosa M. Martí

**Affiliations:** 1 Department of Dermatology, Hospital Universitari Arnau de Vilanova de Lleida, Institut de Recerca Biomèdica de Lleida (IRBLleida), Universitat de Lleida, 25198 Lleida, Spain; marti@medicina.udl.cat; 2 Department of Pathology and Molecular Genetics, Hospital Universitari Arnau de Vilanova de Lleida, Institut de Recerca Biomèdica de Lleida (IRBLleida), Universitat de Lleida, 25198 Lleida, Spain; fvilardell.lleida.ics@gencat.cat (F.V.); avelasco@gss.cat (A.V.); mcuevas@gss.cat (D.C.); msantacana@irblleida.cat (M.S.); sgatius.lleida.ics@gencat.cat (S.G.); fjmatiasguiu.lleida.ics@gencat.cat (X.M.-G.); 3 Tumour Microenvironment, Barts Cancer Institute, Queen Mary University of London, London EC1M 6BQ, UK; o.m.carlos@qmul.ac.uk; 4 Oncological Pathology Group, Institut de Recerca Biomèdica de Lleida (IRBLleida), 25198 Lleida, Spain; carla_barcerlo@medicina.udl.cat (C.B.); pol.siso@udl.cat (P.S.); idelarosa@irblleida.cat (I.d.l.R.); 5 Centre of Biomedical Research on Cancer (CIBERONC), Instituto de Salud Carlos III (ISCIII), 28029 Madrid, Spain; 6 Department of Medical Oncology, Hospital Universitari Arnau de Vilanova de Lleida, Institut de Recerca Biomèdica de Lleida (IRBLleida), Universitat de Lleida, 25198 Lleida, Spain; arodrigo.lleida.ics@gencat.cat; 7 Unitat de Farmacologia- Department of Experimental Medicine, Universitat de Lleida, 25198 Lleida, Spain; amacia@irblleida.cat

**Keywords:** melanoma, *BRAF^V600E^*, next generation sequencing, mutant allele frequency, intratumor heterogeneity

## Abstract

**Simple Summary:**

The mutational load of *BRAF^V600E^* in melanomas has been described as a possible prognostic biomarker but there is no information about the mutant allele frequency (MAF) variability of *BRAF^V600E^* within cutaneous melanomas and its potential prognostic implications. Our study suggests that the variation degree of *BRAF^V600E^* MAF within primary cutaneous melanoma could act as a determinant in the location of primary melanomas as well as their first metastases and could influence prognostic indicators such as Breslow and mitotic indexes. *BRAF^V600E^* MAF variation is also related to the neoplastic cell phenotype and tumor lymphocytic infiltrate of primary tumors. For all these reasons, detection of *BRAF^V600E^* MAF variation could be a useful prognostic biomarker. It is worth exploring the role of *BRAF^V600E^* MAF variation with regards to the response of melanoma to targeted therapies.

**Abstract:**

Background: Cutaneous melanoma shows high variability regarding clinicopathological presentation, evolution and prognosis. Methods: Next generation sequencing was performed to analyze hotspot mutations in different areas of primary melanomas (MMp) and their paired metastases. Clinicopathological features were evaluated depending on the degree of variation of the *BRAF^V600E^* mutant allele frequency (MAF) in MMp. Results: In our cohort of 14 superficial spreading, 10 nodular melanomas and 52 metastases, 17/24 (71%) melanomas had a *BRAF^V600E^* mutation and 5/24 (21%) had a *NRAS^Q61^* mutation. We observed a high variation of *BRAF^V600E^* MAF (H-*BRAF^V600E^*) in 7/17 (41%) MMp. The H-*BRAF^V600E^* MMp were all located on the trunk, had lower Breslow and mitotic indexes and predominantly, a first nodal metastasis. Regions with spindled tumor cells (Spin) and high lymphocytic infiltrate (HInf) were more frequent in the H-*BRAF^V600E^* patients (4/7; 57%), whereas regions with epithelial tumor cells (Epit) and low lymphocytic infiltrate (LInf) were predominant (6/10; 60%) and exclusive in the low *BRAF^V600E^* MAF variation tumors (L-*BRAF^V600E^*). The H-*BRAF^V600E^*/Spin/HInf MMp patients had better prognostic features and nodal first metastasis. Conclusions: The H-*BRAF^V600E^* MMp were located on the trunk, had better prognostic characteristics, such as lower Breslow and mitotic indexes as well as high lymphocytic infiltrate.

## 1. Introduction

Cutaneous melanoma (CMM) is the most aggressive skin malignancy. Although CMM can be cured with wide local excision when it is diagnosed in the early stages, advanced melanoma is associated with poor outcomes [1]. Nevertheless, knowledge advances in melanoma pathogenesis and the subsequent development of targeted and immunobased therapies have improved metastatic melanoma treatment [2].

The MAPK–ERK pathway plays a major role in the development and progression of melanoma [3]. Overall, about 35–50% of melanomas of all clinical types have mutations in the V600 codon of *BRAF* [4]. Approximately 90% of *BRAF* mutations result in the substitution of valine (V) for glutamic acid (E) at position 600 (*BRAF^V600E^*) [5,6,7,8,9]. The *BRAF^V600E^* mutation confers to this kinase the ability to activate MEK [4,10]. This ligand-independent pathway activation is essential in melanoma development and progression [11,12]. Combinations of BRAF and MEK inhibitors have emerged as new treatment options for patients with *BRAF*-mutated melanoma with a good response rate [13,14]. Unfortunately, most patients that initially show an extremely good response to BRAF inhibitors develop resistance to those drugs within a relatively short period of time (<1 year) [10,14].

Besides the usefulness of the *BRAF^V600E^* driver mutation as a therapeutic target, a recent study has suggested that the mutational load of *BRAF^V600E^* is a possible prognostic biomarker [15], expanding the classical histopathological prognostic criteria such as ulceration, Breslow, Clark or mitotic indexes [1,16,17,18,19,20,21,22]. Moreover, some studies in recent years have attempted to relate the mutational load to the response in order to target therapies, but controversial results have been obtained [23,24,25,26].

Next-generation sequencing (NGS) technologies have played an essential role in understanding the altered genetic pathways involved in human cancer. It is a high-throughput method which allows for the sequencing of multiple samples and targeted genes in the same run. NGS can quantitate the proportion of reads for a given mutation, also known as mutant allele frequency (MAF), which represents the percentage of tumor cells that harbor a specific mutation in a neoplastic tissue. The sensitivity of NGS is higher than that observed with Sanger sequencing (2–10% versus 15–25% allele frequency, respectively) [27,28].

It is widely accepted that melanomas are compound by tumor cells, with different histophenotypic characteristics (morphology or pigmentation), and different non-tumor cells as inflammatory cells. These characteristics can also be different between the primary tumor and its metastases [29]. Therefore, understanding MAF using NGS technology could provide invaluable information regarding melanomagenesis and dissemination.

In this work, we used NGS to study the variation of *BRAF^V600E^* MAF between different regions of 24 primary melanomas and their paired metastases as well as its relation to their histophenotypic and clinical characteristics, in an attempt to identify the predictors of disease outcome.

## 2. Materials and Methods

### 2.1. Patient Selection and Data Collection

The Pathology Department archive was retrospectively reviewed from 1 January 2000 to 30 June 2020. Patients who had paired primary cutaneous melanoma (MMp) and metastases (MMx) were selected. In order to avoid excessive genetic diversity, the study was restricted to superficial spreading melanoma (SSM) and nodular melanoma (NM), according to the WHO classifications [8].

Gender, age, MMp subtype, Breslow and Clark indexes, presence of ulceration, number of mitoses, MMp location, number and location of the MMx, disease free survival (DFS) and overall survival (OS) were recorded.

### 2.2. Diagnostic Criteria of Histological Features

**Figure 1:**
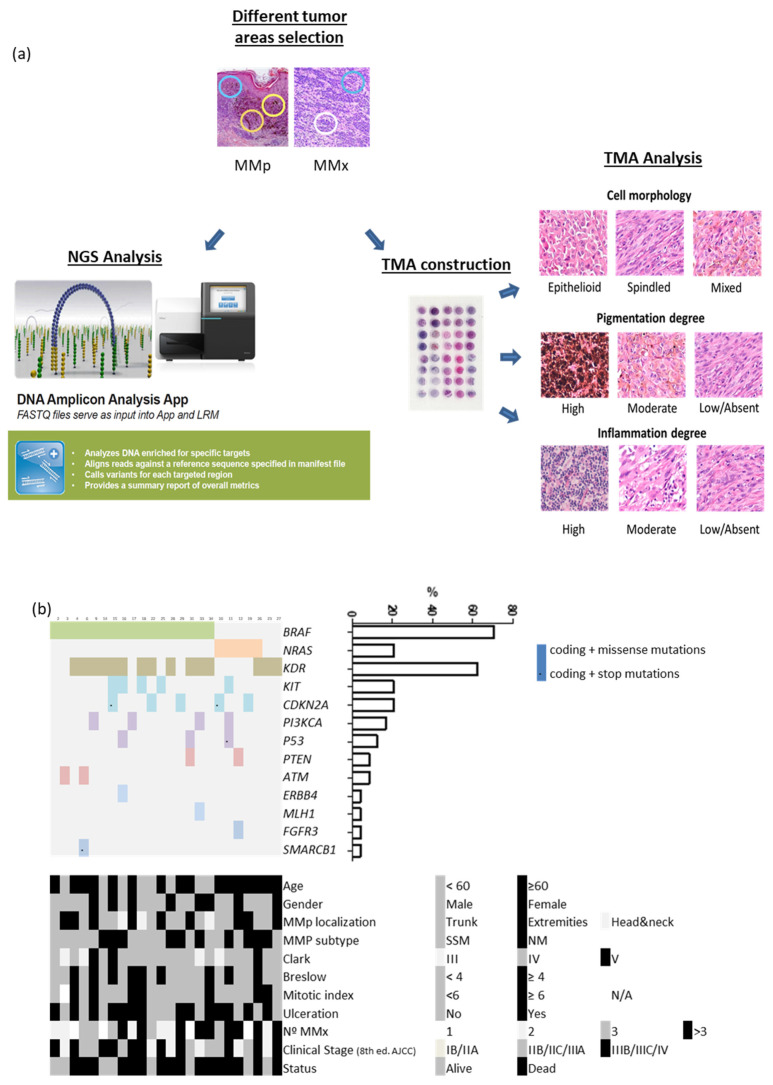
(**a**) Study workflow design. (**b**) Heatmap showing mutated genes, demographic, clinical and tumor characteristics of our patient cohort.

A detailed microscopic analysis of routine H&E sections for the identification of areas of different cell morphology (spindled, epithelioid and mixed) and different degree of pigmentation (absent/low, moderate and high) was performed. A selection of 3 and 2 different areas of the MMp and the MMx, respectively, was done (Figure 1a).

### 2.3. DNA Extraction and NGS Analysis

In total, 191 areas selected were microdissected from the formalin-fixed paraffin-embedded blocs. DNA extracted by Maxwell^®^ FFPE Plus DNA Kit (Promega, Madison, WI, USA) were quantified by Qubit fluorometer (Thermo Fisher Scientific, Waltham, MA, USA).

We performed NGS with the Ampliseq Cancer HotSpot panel v2 (Illumina, San Diego, CA, USA) to detect genetic mutations from 50 oncogenes and tumor suppressor genes. Genomic DNA (10 ng) was used to construct the amplicon libraries according to the manufacturer’s protocol and quality control of the libraries was done with Agilent 2100 Bioanalyzer. Resulting data files were analyzed by DNA amplicon analysis workflow to perform alignment and variant calling. We excluded non-coding variants and synonymous changes of each polymorphism. Single nucleotide variants with a variant allele frequency of at least 10% and a minimum depth coverage of 150 reads were used as cut-off values for variant identification. (Figure 1a).

### 2.4. TMA Elaboration and Analysis

Four tissue microarrays (TMAs) containing the 191 areas, were constructed using a tissue arrayer device (Beecher Instrument, Sun Prairie, WI, USA). Histopathological tumor characteristics (cell morphology, degree of pigmentation and lymphocytic infiltrate) were qualitatively analyzed within each TMA spot. Cell shape was visually graded as epithelioid or spindled when the majority of the tumor cells expressed the same phenotype (>80) and mixed when both phenotypes were observed and none of them were >80%.

Pigmentation was scored on a three-point scale (high, moderate and low/absent). Melanophages were not considered. Lymphocytic infiltrate was scored on a three-point scale (high, moderate and low/absent). The dissected areas contained at least 50% of tumor nuclei. (Figure 1a).

#### Immunohistochemical Analysis of *BRAF^V600E^* Specific Target Protein

TMA *BRAF^V600E^* immunostaining was performed with the VE1 anti-*BRAF^V600E^* primary antibody (Ventana Medical Systems, Inc., Roche, Basel, Switzerland) as described [30]. Staining quantification was performed with QuPath 0.2.3 software (Centre for Cancer Research & Cell Biology, Queen’s University Belfast, UK). Values ranged from 0 to 300 [31].

### 2.5. Statistical Analysis

The Fisher exact test and T-test were used to examine associations between high or low intratumoral *BRAF^V600E^* MAF variation (H-*BRAF^V600E^* or L-*BRAF^V600E^*) and demographic, clinical and MMp histopathological variables. Overall survival (OS) and disease-free survival (DFS) curves were calculated using the Kaplan–Meier method with log-rank test. The Mann–Whitney test was used to assess immunohistochemical characteristics. The *p*-values are indicated by asterisks * *p* < 0.05; ** *p* < 0.01; *** *p* < 0.001. All statistical analyses and figures were performed using SPSS Statistics software and Graphpad Prism software.

## 3. Results

### 3.1. Clinicopathological Characteristics

Our series comprised 29 patients with 29 MMp and 52 paired MMx, which supposed a total number of 87 MMp and 104 MMx spots. Due to low quality samples, 5 patients were rejected and 24 patients were elected for further analysis (Figure S1). This cohort was composed of 16 (67%) men and 8 (33%) women, with a median age of 63 years (24–87). The MM subtype was SSM in 14 (58%) and NM in 10 (42%) biopsies. In total, 12 (50%) MMp were located on the trunk, 6 (25%) on the lower extremities, 3 (12.5%) on the upper extremities and 3 (12.5%) on the head and neck. The mean MMp Breslow and Clark indexes were 4.22 mm (1.04–13.50) and 4 (3–5), respectively. Ulceration was present in 14 (58%) cases and the mean mitotic index was 5/cm^2^ (1–11). Eight patients (33.33%) were alive at the end of the study with a mean survival time of 60 months (median 40.5 (9–177)) (Table 1 and Table S1).

We gathered the NGS results and pathological characteristics of 94 spots (66 MMp and 28 MMx spots), corresponding to 24 MMp and 15 paired MMx (Figure 1a). We did not observe significant differences in the distribution of pathological characteristics between the MMp and MMx groups (Table S2).

### 3.2. Genetic Analysis: There Is Variation in BRAF^V600E^ Mutant Allele Frequency in Primary Melanomas

NSG of 66 MMp and 28 MMx regions identified mutations in seven oncogenes (*BRAF, NRAS, KIT, KDR, FGFR3, ERBB4* and *PI3KCA*) and six tumor suppressor genes (*CDKN2A, TP53, ATM, PTEN, MLH1* and *SMARCB1*) in the MMp (Figure 1b). The *BRAF^V600E^* mutation was present in 17/24 (71%) patients, *NRAS^Q61^* in 5/24 (21%) and 2/24 (8%) MMp were of the wild type for both genes. We compared the clinicopathological characteristics of *BRAF^V600E^* and *NRAS^Q61^* patients and no statistical differences were detected. The *BRAF^V600E^* and *NRAS^Q61^* mutations where highly preserved in the MMp and their paired MMx. Only 2/17 (11.8%) *BRAF^V600E^* mutated patients presented an absence of *BRAF^V600E^* mutation in one of the three MMp spots (intratumor heterogeneity). Additionally, in 2/17 (11.8%) patients whose three MMp spots were *BRAF^V600E^* mutated, both spots of their paired MMx were the *BRAF* wild type (intertumor heterogeneity) [32,33]. Across the cohort, 11.8% intratumoral and intertumoral heterogeneity was detected in the *BRAF^V600E^* mutation. 

**Figure 2:**
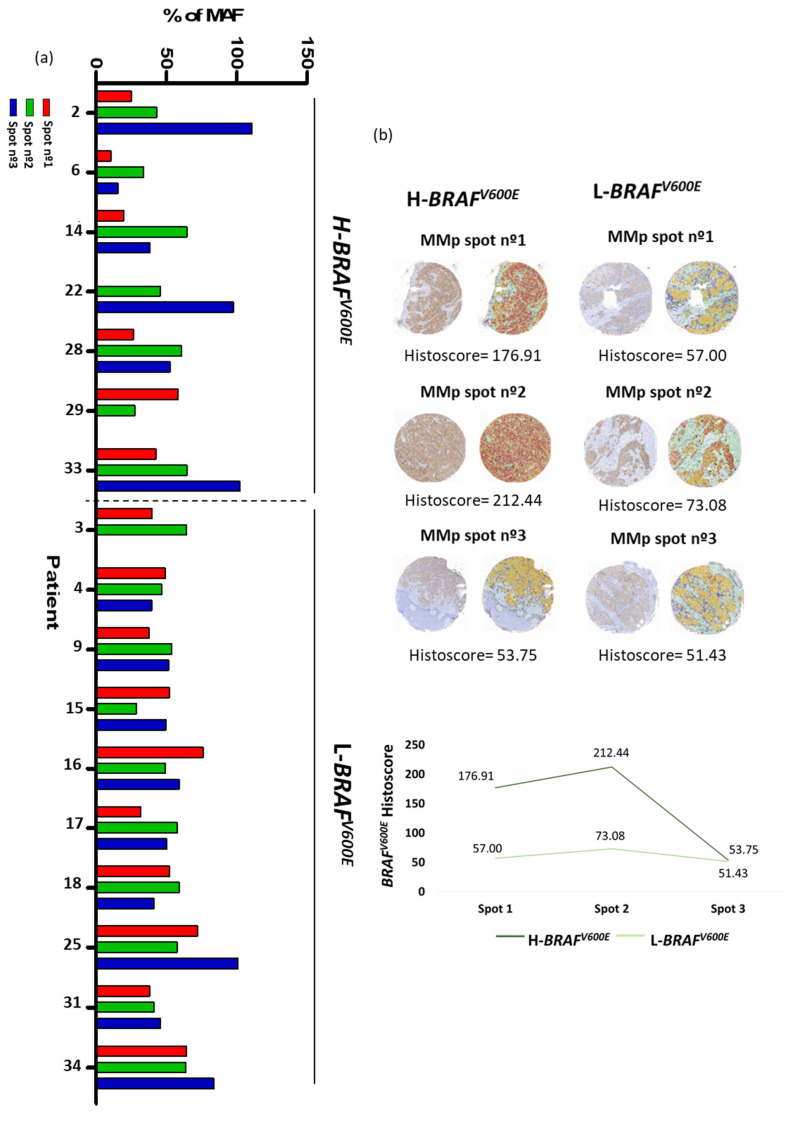
MAF of *BRAF^V600E^* (**a**) NGS analysis of *BRAF^V600E^* MAF corrected by % of tumor cells. Patients grouped in H-*BRAF^V600E^* and L-*BRAF^V600E^*. (**b**) Immunohistochemical examples of primary melanoma tumor with high immunohistochemical histoscore variation of *BRAF^V600E^* between its spots and primary melanoma tumor with low immunohistochemical histoscore variation of *BRAF^V600E^* between its spots.

Striking differences in the MAF of *BRAF^V600E^* between spots of the same MMp were detected. Therefore, we decided to quantify such variations. First, to obtain the real *BRAF^V600E^* MAF we took into account the percentage (%) of tumoral cells present in each spot by three investigator-blinded assessments on H&E staining. Discrepant cases were discussed until an agreement was reached. Only spots composed of at least 50% tumor cells were considered. In all cases, the mean *BRAF^V600E^* MAF obtained by NGS was recalculated depending on the percentage of tumor cells observed in each spot from the TMA created. We defined H-*BRAF^V600E^* primary tumors as those that contained one spot with a two-fold increase MAF value regarding at least one of the other MMp spots. Samples that lacked this diversity were considered L-*BRAF^V600E^*. In our cohort of 17 MMp with the *BRAF^V600E^* mutation, 7 (41.18%) showed H-*BRAF^V600E^* while the remaining 10 (58.82%) had L-*BRAF^V600E^* (Figure 2a).

### 3.3. Immunohistochemical Analysis: There Is Variation in BRAF^V600E^ Immunoexpression in Primary Melanomas

High and low variation in *BRAF^V600E^* MAF was validated by an immunohistochemical study.

First, histoscores of *BRAF^V600E^* were obtained by Qupath analysis from all of the TMA spots. As expected, *NRAS* mutated MMp spots presented a low histoscore (<20). Then, we considered a histoscore of ≥20 as the cut-off for the immunohistochemical histoscore scale for *BRAF^V600E^* positivity with a sensitivity of 87.5% and a specificity of 100% [34,35,36].

Then, we defined tumors with high immunohistochemical variation of *BRAF^V600E^* as those primary tumors that contained one spot with a two-fold increase histoscore value regarding at least one of the other MMp spots. Samples that lacked this diversity were considered to show a low immunohistochemical variation of *BRAF^V600E^* (Figure 2b). We observed that immunohistochemical evaluation of *BRAF^V600E^* showed significant consistency with the NGS analysis, as it was able to identify patients with H-*BRAF^V600E^* with a sensitivity of 80% and a specificity of 77.78%.

### 3.4. Correlation between BRAF^V600E^ Allelic Frequency Variation and Histomorphological Features

#### 3.4.1. H-*BRAF^V600E^* Correlation with Demographic, Clinical and Tumor Characteristics

**Figure 3:**
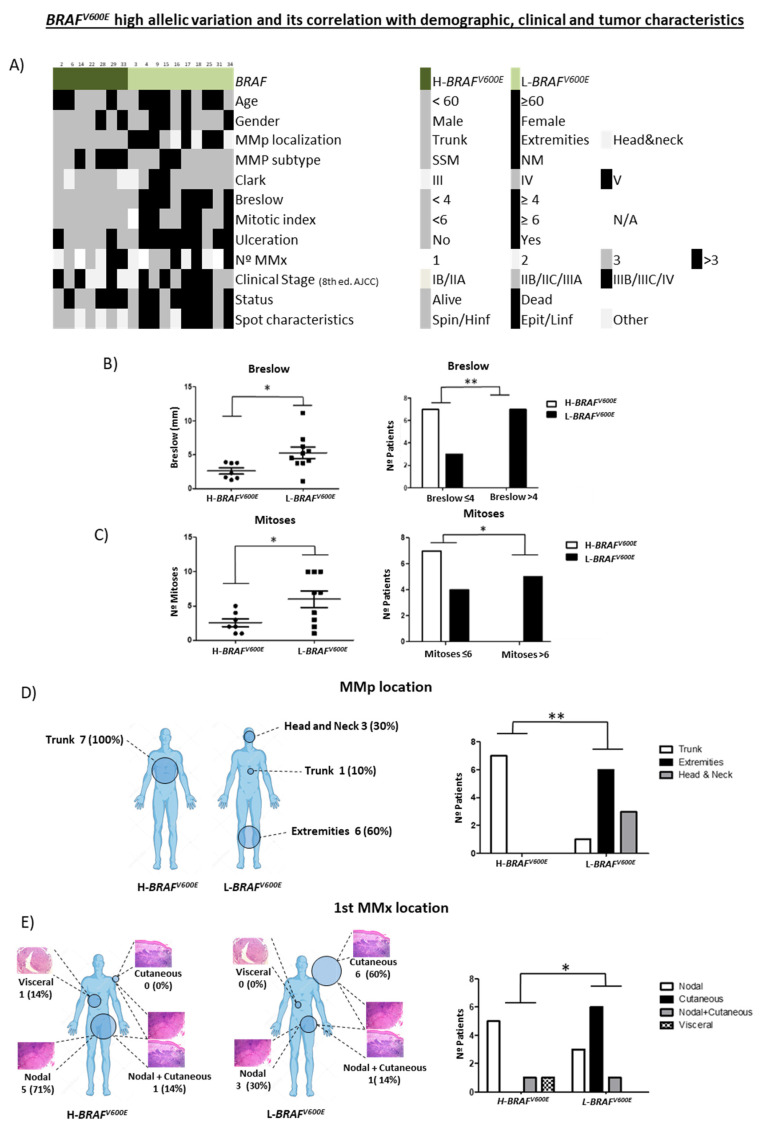
Clinicopathological characteristics of MMp depending on *BRAF^V600E^* MAF variation (high vs. low). (**A**) Heatmap showing demographic, clinical and tumor characteristics of MMp patients depending on *BRAF^V600E^* MAF variation. (**B**) Breslow thickness depending on *BRAF^V600E^* MAF variation and number of patients according to Breslow thickness (<4 mm vs. ≥4 mm) in primary melanoma tumors. (**C**) Number of mitoses and number of patients according to mitotic index (<6 vs. ≥6) depending on *BRAF^V600E^* MAF variation. (**D**) MMp and (**E**) first MMx location depending on H-*BRAF^V600E^* vs. L-*BRAF^V600E^* patients. Statistical analysis was performed using *t*-test, Fischer exact tests (* *p* < 0.05; ** *p* < 0.01).

First, we analyzed all clinicopathological characteristics of H-*BRAF^V600E^* patients compared with those of the L-*BRAF^V600E^* group (Figure 3A).

H-*BRAF^V600E^* patients had statistically lower Breslow and mitotic indexes than L-*BRAF^V600E^* (Mean 2.617 ± 0.4426 vs. 5.280 ± 0.8373 and Mean 2.571 ± 0.5714 vs. 6.000 ± 1.202). These results indicated that the H-*BRAF^V600E^* MMp had better prognostic features than the L-*BRAF^V600E^* since their Breslow tended to be <4 mm and they had a lower mitotic index [1,20,22] (Figure 3B,C). No statistical differences were observed regarding age and gender, MMp subtype, Clark or ulceration.

In addition, we analyzed the MMp and MMx locations in both groups. H-*BRAF^V600E^* patients presented all their MMp on the trunk (7/7 100%), while L-*BRAF^V600E^* patients presented most of their MMp in other locations, mainly on the extremities (6/10; 60%) but also on the head and neck (3/10; 30%) and trunk (1/10; 10%) (Figure 3D).

Moreover, we observed important differences in the first MMx location. Most H-*BRAF^V600E^* patients presented a nodal first MMx (6/7; 86%), while the first MMx in L-*BRAF^V600E^* patients was mostly cutaneous/subcutaneous (7/10; 70%) (Figure 3E). Interestingly, in H-*BRAF^V600E^* patients the nodal MMx was detected in the sentinel lymph node biopsy in 4/6 (67%) cases, whereas in 4/7 (57%), skin MMx of the L-*BRAF^V600E^* patients were already present at diagnosis of the primary tumor as a satellitoses or in-transit metastases. No statistical differences were observed in OS and DFS between the groups (Figure S2a).

All these results indicate that *H-BRAF^V600E^* seems to correlate with the development of the MMp on the trunk, better prognostic features such as lower Breslow and mitotic indexes, and nodal first MMx.

#### 3.4.2. Demographic, Clinical and Tumor Characteristics of H-*BRAF^V600E^*/Spindled/ High Lymphocytic Infiltrate Group vs. L-*BRAF^V600E^*/Epithelioid/Low Lymphocytic Infiltrate Group

**Figure 4:**
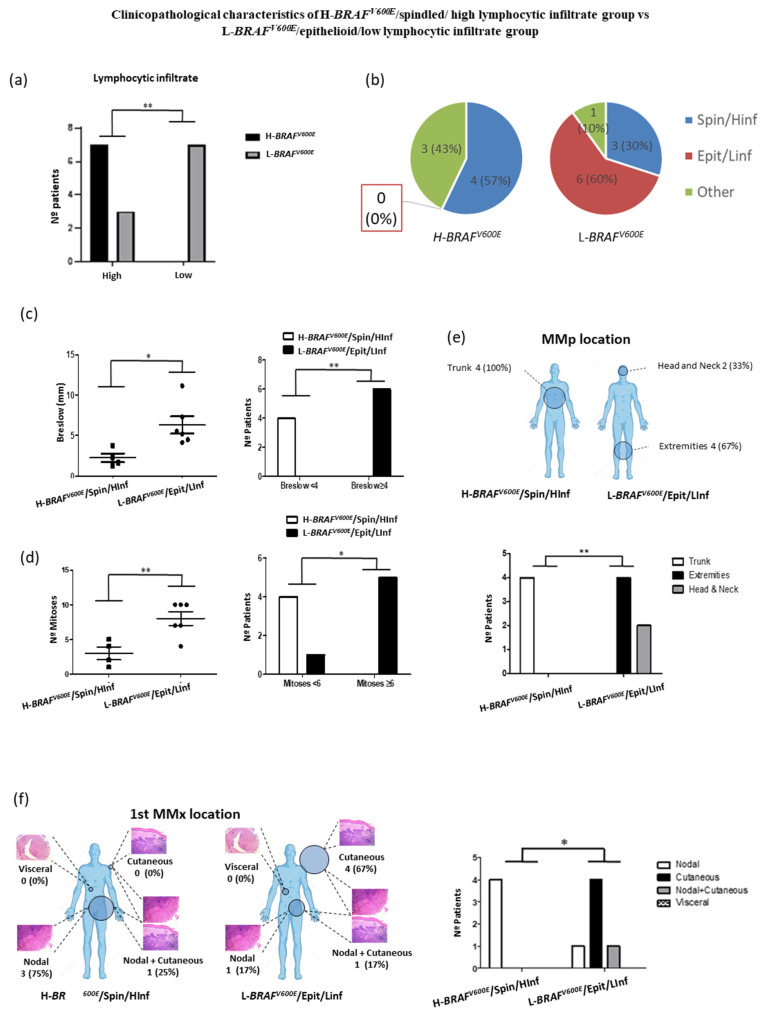
(**a**) Patient distribution according to variation of *BRAF^V600E^* MAF (high vs. low) and lymphocytic infiltrate degree of their primary melanoma spots. (**b**) Patient distribution according to variation of *BRAF^V600E^* MAF and the combination of their histophenotypic characteristics and lymphocytic infiltrate degree of the primary melanoma spots. (**c**) Breslow thickness depending on variation of *BRAF^V600E^* MAF, cytological characteristics and lymphocytic infiltration degree and number of patients according to Breslow thickness (<4 mm vs. ≥4 mm) in primary melanoma tumors. (**d**) Number of mitoses and number of patients according to mitotic index (<6 vs. ≥6) depending on variation of *BRAF^V600E^* MAF, cytological characteristics and lymphocytic infiltration degree. (**e**) MMp and (**f**) first MMx location depending on H-*BRAF^V600E^*/Spin/HInf vs. L-*BRAF*^V600E^/Epit/LInf patients. Statistical analysis was performed using *t*-test and Fischer exact tests (* *p* < 0.05; ** *p* < 0.01).

We wanted to assess if the variation in *BRAF^V600E^* MAF was correlated with a certain cytological morphology, pigmentation or lymphocytic infiltrate degree. We identified that 7/7 (100%) H-*BRAF^V600E^* patients had at least one spot with high-moderated lymphocytic infiltrate (HInf) while 7/10 (70%) L-*BRAF^V600E^* patients had low lymphocytic infiltrate (LInf) in all their spots (Figure 4a). Moreover, the melanoma cells in 4/7 (57%) H-*BRAF^V600E^* patients with the HInf spot had a spindled phenotype while L-*BRAF^V600E^* patients with the HInf spot and spindled phenotype were observed in 3/10 (30%) patients. On the other hand, 6/10 (60%) L-*BRAF^V600E^* patients had all LInf spots and their melanoma cells were epithelioid, while none of the H-*BRAF^V600E^* patients had this late characteristic (0%) (Figure 4b). Therefore, we divided the patients into two groups: patients who presented H-*BRAF^V600E^* and had at least one spindled and high-moderated lymphocytic infiltrate spot (H-*BRAF^V600E^*/Spin/HInf) vs. patients with L-*BRAF^V600E^* and epithelioid and low lymphocytic infiltrate (L-*BRAF^V600E^*/Epit/LInf) spots.

The H-*BRAF^V600E^*/Spin/HInf group showed a significantly lower Breslow and mitotic index than the L-*BRAF^V600E^*/Epit/Linf group (Mean 2.270 ± 0.5533 vs. 6.308 ± 1.067 and Mean 3.000 ± 0.9129 vs. 8.000 ± 1.000) (Figure 4c,d). H-*BRAF^V600E^*/Spin/HInf patients presented all their MMp on the trunk (4/4; 100%) while L-*BRAF^V600E^*/Epit/LInf patients presented all their MMp on the extremities (4/6; 67%) and head and neck (2/6; 33%) (Figure 4e). There were no statistical differences in age, gender, MMp subtype, Clark index, presence of ulceration or number of MMx. Nevertheless, we observed differences in the first MMx location. All H-*BRAF^V600E^*/Spin/HInf patients had a nodal MMx (4/4; 100%) whereas 5/6 (83.33%) L-*BRAF^V600E^*/Epit/Linf patients had a cutaneous first MMx (Figure 4f). Interestingly, in H-*BRAF^V600E^* patients the nodal MMx was detected by sentinel lymph node biopsy in 3/4 (75%) cases, whereas 5/5 (100%) skin MMx of the L-*BRAF^V600E^* patients were present at MMp diagnosis as a satellitoses or in-transit metastases. No statistical differences were observed in OS and DFS between the groups (Figure S2b).

All these results suggest that the spindle phenotype and high/moderate lymphocytic infiltrate correlate with the *H-BRAF^V600E^* samples. This confers better clinicopathological prognostic characteristics for these MM patients, such as lower Breslow and mitotic indexes [1,20,22].

## 4. Discussion

Melanoma can show different morphological characteristics such as different cytological subtypes or degrees of pigmentation either between different MMp or even within the same MMp (intratumoral heterogeneity) and between the MMp and paired MMx (intertumoral heterogeneity).

This study attempts to investigate the relationship between the diverse histophenotypic features present in different regions of MMp compared with the genetic characteristics found by NGS assay in a series of 24 patients with primary SSM or NM.

We detected mutations in seven oncogenes (*BRAF, NRAS, KIT, KDR, FGFR3, ERBB4* and *PI3KCA*) and six tumor suppressor genes (*CDKN2A, TP53, ATM, PTEN, MLH1* and *SMARCB1*) in our MMp series. Other studies using NGS and cancer hotspot panels in paired melanoma samples found the same main driver mutations (*BRAF, NRAS, KIT*) [29,37]. However, none of these studies analyzed the *BRAF^V600E^* MAF variation.

*BRAF* and *NRAS* mutation frequencies differ between cutaneous melanoma subtypes. *BRAF* mutation occurs more frequently in SSM (20–65%) and in second place in NM (20–43%) [38,39,40,41,42,43], while NM is the most frequent MM subtype with *NRAS* mutations (21–27%) [41,44]. Our series only included SSM and NM, which harbored the *BRAF^V600E^* mutation in 71% and *NRAS^Q61^* in 21% of MMp. These findings were similar to those previously described, although the presence of *BRAF^V600E^* was slightly higher. As the number of SSM and NM in our series are quite balanced (14 and 10 cases, respectively) differences in sequencing methodology could perhaps explain this fact, since a greater sensibility of NGS has been described in the detection of *BRAF* mutations compared to allele specific PCR [37,45].

We found that the *BRAF^V600E^* and *NRAS^Q61^* mutations where highly preserved in MMp and their paired MMx. Only 2/17 (11.8%) cases presented intratumor heterogeneity and 2/17 (11.6%) cases presented intertumor heterogeneity regarding *BRAF^V600E^* mutation. These findings support the idea that driver mutations occur early during the development of malignant melanoma [23,38,39,46]. Then, melanoma can show intratumoral and intertumoral *BRAF^V600E^* mutation heterogeneity, although its frequency is low.

In recent years, controversial results have been obtained by studies which related the *BRAF^V600E^* MAF of melanoma tumors with clinicopathological and prognostic characteristics as well as the melanoma response, in order to target therapies [15,23,24,25,26]. All these studies analyzed the MAF value of one specific region for each tumor, so we wanted to know whether the variation degree of *BRAF^V600E^* MAF within different regions of the same tumor could influence these characteristics.

Interestingly, our study suggests that variation of *BRAF^V600E^* MAF within MMp is related to the clinical features of the MMp, histopathological prognostic factors and progression behavior, although all the patients in our series developed metastasis at some point (since it was one of the inclusion criteria). Thus, H-*BRAF^V600E^* MMp would settle more frequently on the trunk and their first MMx would more frequently be nodal, while the L-*BRAF^V600E^* melanomas would be located more frequently on the extremities and develop most often cutaneous/subcutaneous, regional first MMx. The preferential location of the first MMx on the skin in L-*BRAF^V600E^* MMp cases could be explained because all were satellite or in-transit MMx, and this type of MMx are more frequently associated with MMp located on the extremities [47,48]. In addition, the H-*BRAF^V600E^* patients seem to have better histopathological prognostic indicators such as lower Breslow and mitotic indexes [1]. These associations were even more evident when we analyzed the H-*BRAF^V600E^*/Spin/Hinf and L-*BRAF^V600E^*/Epit/Linf subgroups. We identified an even stronger predilection of the MMp and first MMx for the trunk and nodal regions, respectively, with lower Breslow and mitotic indexes in the H-*BRAF^V600E^*/Spin/Hinf patients, while the L-*BRAF^V600E^*/Epit/Linf MMp were preferentially located on the extremities and their first MMx was significantly more frequently cutaneous, showing higher Breslow and mitotic indexes. Our findings could be explained in part by the suggestion of some authors that the presence of tumor-infiltrating lymphocytes (TILs) in melanoma is associated with a lower Breslow and mitotic rate [49,50,51]. Moreover, with regards to cytological features, a recent study showed that melanoma cells with rounded behavior had a higher capacity for tumor formation, tumor progression and metastasis than elongated cells [52], indicating that an increase in epithelioid cells in MMp could mean a higher proliferative and invasive behavior.

Even though we are aware of the modest sample size, our results suggest that variation of *BRAF^V600E^* MAF can constitute a potential biomarker that is worth exploring further. Moreover, these findings could serve as initial step for the future development of therapies focused on the presence of worse genetic and histopathological characteristics, such as L-*BRAF^V600E^*, epithelioid cell compound and low lymphocytic infiltrate in MMp.

## 5. Conclusions

The attained results suggest that MAF variation of *BRAF^V600E^* within primary cutaneous melanomas (MMp) could influence their histopathological characteristics, as well as their anatomic location and that of their first metastases (MMx). Here we described for the first time in the biomedical literature, that H-*BRAF^V600E^* could correlate with a spindle phenotype, high/moderate lymphocytic infiltrate and the development of the MMp on the trunk and nodal first MMx. On the contrary, L-*BRAF^V600E^* seems to correlate with an epithelioid phenotype, low lymphocytic infiltrate, the development of the MMp predominantly on the extremities and a cutaneous first MMx.

In addition, we suggest a possible new link between H-*BRAF^V600E^* and better classical prognostic indicators such as lower Breslow and mitotic indexes.

## Figures and Tables

**Table 1:** Clinicopathological Characteristics.

Gender	Number (Percentage/Range)
Male	16 (67%)
Female	8 (33%)
**Median age**	63yo (24–87)
**MM subtype**	
SSM	14 (58%)
NM	10 (42%)
**MMp location**	
Trunk	11 (50%)
Lower extremities	6 (25%)
Upper extremities	3 (12.5%)
Head and neck	3 (12.5%)
**Mean Breslow**	4.22 mm (1.04–13.50)
**Mean Clark**	4 (3–5)
**Ulceration**	
Yes	14 (58%)
No	10 (42%)
**Mean mitotic index**	5 mitoses/cm^2^ (1–11)
**MMx location**	
Cutaneous	27 (51.92%)
Nodal	13 (25%)
CNS	3 (5.77%)
Lung	2 (3.85%)
Suprarenal	2 (3.85%)
Muscular	2 (3.85%)
Parotid	1 (1.92%)
**Live status**	
Alive	8 (33.3%)
Dead	16 (66.7)
**Mean survival**	69 months (median 40.5 (9–177))

SSM: superficial spreading melanoma; NM: Nodular melanoma; CNS: Central nervous system.

## Data Availability

The data presented in this study are available in this article (and Supplementary material).

## Supplementary Materials

The following are available online at https://www.mdpi.com/article/10.3390/cancers13205073/s1, Figure S1: Patient selection; Figure S2: (a) Overall survival and disease free survival of patients depending on BRAFV600E MAF variation (high vs. low); (b) Overall survival and disease free survival of H-*BRAF^V600E^*/Spin/HInf vs. L-*BRAF^V600E^*/Epit/Linf patients.; Table S1: Demographic, clinical and tumor characteristics of our series.; Table S2: Histophenotypic sample characteristics.
