# Supplementary material for: BRAFV600E Mutant Allele Frequency (MAF) Influences Melanoma Clinicopathologic Characteristics
Source: Cancers (Basel). 2021 Oct 11;13(20):5073. doi: 10.3390/cancers13205073 (PMC8533792; doi:10.3390/cancers13205073)
Supplement: Supplementary file 1 [file cancers-13-05073-s001.zip › cancers-1382725 supplementary after final.pdf]

cancers

MDPI

Supplementary Material: *BRAF*^{V600E} Mutant Allele Frequency (MAF) Influences Melanoma Clinicopathologic Characteristics

Xavier Soria, Felip Vilardell, Óscar Maiques, Carla Barceló, Pol Sisó, Inés de la Rosa, Ana Velasco, Dolors Cuevas, Maria Santacana, Sònia Gatius, Xavier Matías-Guiu, Alberto Rodrigo, Anna Macià and Rosa M. Martí

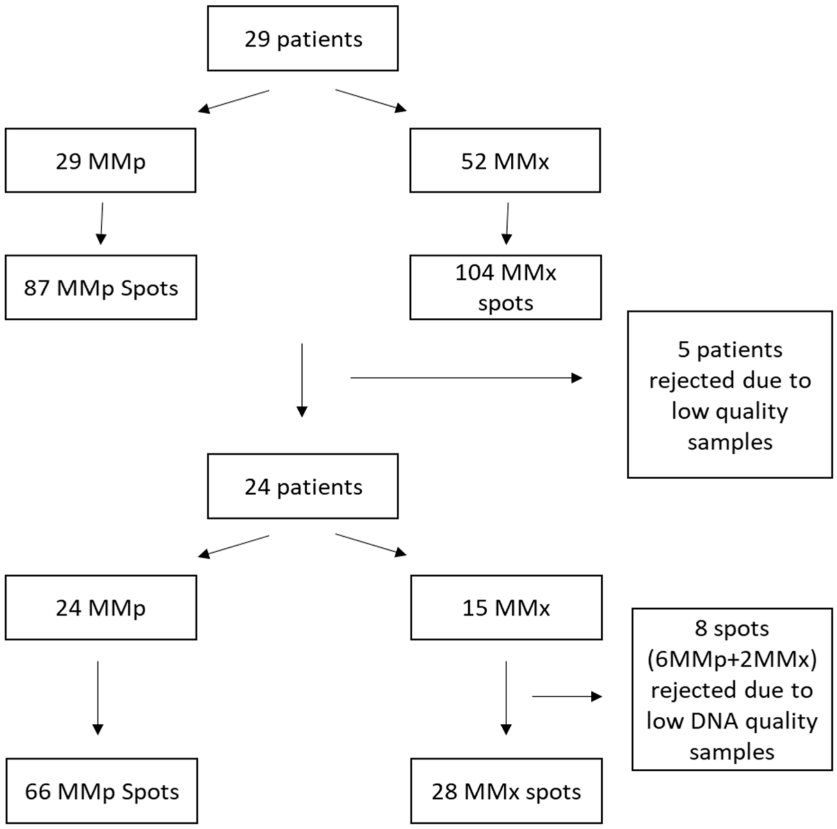

Figure S1. Patient selection.

(a)

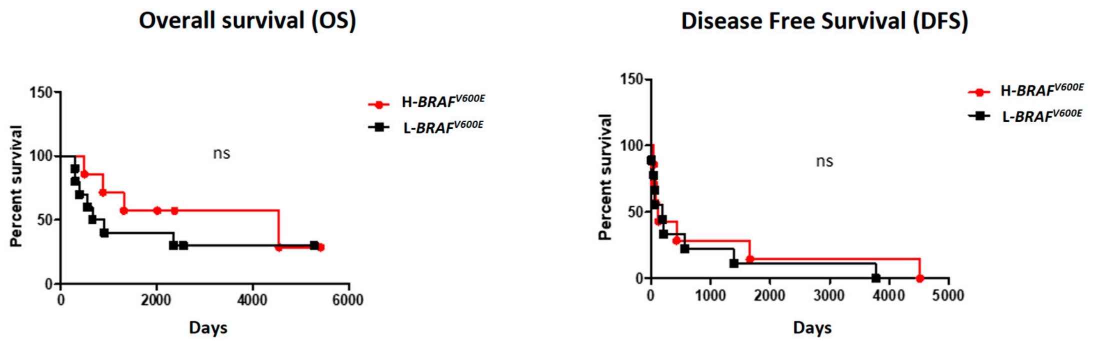

(b)

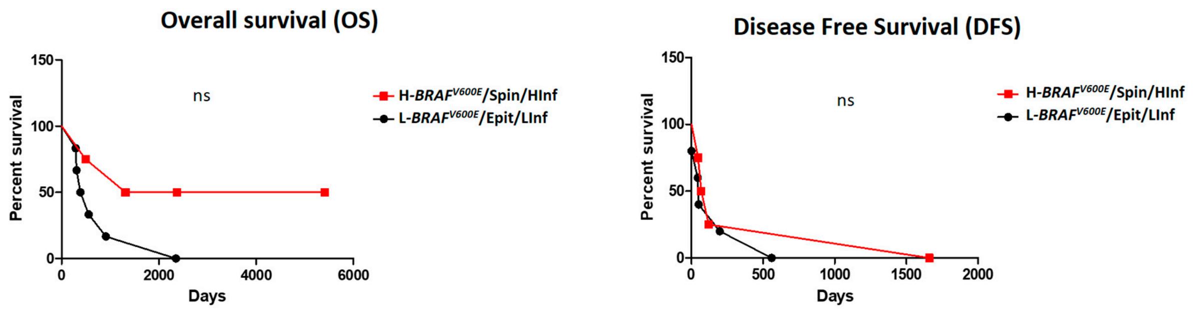

Statistical analysis was performed using the Kaplan-Meier method. (ns: non-significant)

Figure S2. (**a**) Overall survival and disease free survival of patients depending on BRAFV600E MAF variation (high vs. low); (**b**) Overall survival and disease free survival of H-*BRAF*^{V600E}/Spin/HInf vs. L-*BRAF*^{V600E}/Epit/Linf patients.

Table S1. Demographic, clinical and tumor characteristics of our series.

Case nº	Gender	Age*	MMp subtype	Location	Breslow index	Clark index	Ulceration	Mitotic index	Driver mutation	MMx subtype	Treatment	Dead (months)**	MM as cause of death
**1**	M	63	SSM	Trunk	3,81	4	Yes	1	$BRAF^{V600E}$	Nodal (regional)<br>Visceral	SE+RT (adjuvant)<br>Verumafenib	No (77)	-
**2**	M	68	SSM	Trunk	1,30	3	No	2	$BRAF^{V600E}$	Nodal (regional)<br>Visceral	SE+RT (adjuvant)<br>Palliative	Yes (43)	Yes
**3**	M	58	NM	Trunk	3,77	4	No	2	$BRAF^{V600E}$	Nodal (regional)	SE + IFN (adjuvant)	No (66)	-
**4**	F	61	NM	Trunk	6,23	5	Yes	2	$BRAF^{V600E}$	Nodal (regional)<br>Skin(in-transit/distant)	SE+RT (adjuvant)<br>Encorafenib + Binimetinib	No (77)	-
**5**	M	37	SSM	Trunk	1,67	4	No	5	$BRAF^{V600E}$	Nodal (regional)<br>Skin (distant)	SE + RT (adjuvant)<br>Ipilimumab (1st line);<br>Verumafenib (2nd line)	No (177)	-
**6**	M	66	SSM	Extremities	5,20	4	Yes	10	$BRAF^{V600E}$	Skin (satellitosis)	SE	Yes (9)	No
**7**	F	85	SSM	Extremities	4,15	4	Yes	7	$BRAF^{V600E}$	Skin (satellitosis)<br>Nodal (regional)	SE	Yes (12)	No
**8**	M	46	SSM	Extremities	1,12	3	No	N/A	$BRAF^{V600E}$	Skin (in-transit)	Nivolumab	No (173)	-
**9**	F	86	SSM	Extremities	7,30	5	Yes	7	$BRAF^{V600E}$	Skin (in-transit)<br>Nodal (regional)	IC<br>PT	Yes (29)	Yes
**10**	M	58	NM	Head and neck	3,80	4	Yes	1	$BRAF^{V600E}$	Nodal (regional) -<br>Visceral	SE+ RT (adjuvant)<br>Verumafenib	Yes (21)	Yes
**11**	M	24	SSM	Head and neck	4,50	4	Yes	10	$BRAF^{V600E}$	Skin (satellitoses/in-<br>transit)<br>Visceral	SE+RT<br>Nivolumab (1st line)<br>Dabrafenib+<br>Trametinib (2nd line)	Yes (18)	Yes
**12**	M	69	NM	Trunk	5,01	4	Yes	4	$NRAS^{Q61}$	Nodal (regional) -<br>visceral	SE + RT<br>PT	Yes (9)	Yes
**13**	F	81	SSM	Extremities	1,28	4	No	1	$NRAS^{Q61}$	Skin (in-transit)<br>Visceral	IC<br>PT	Yes (24)	Yes
**14**	M	63	NM	Extremities	3,88	4	Yes	2	$NRAS^{Q61}$	Nodal (regional)	SE + IFN<br>(adjuvant)	No (91)	-
**15**	M	63	NM	Trunk	13.50	5	No	11	$NRAS^{Q61}$	Visceral<br>Skin (regional)	IQ	Yes (38)	Yes
**16**	M	70	NM	Trunk	1.90	4	Yes	8	WT	Visceral	SE	No (146)	-
**17**	F	87	SSM	Extremities	2.17	4	No	2	WT	Skin (in-transit)<br>Nodal (regional)	IC<br>PT	Yes (34)	Yes
**18**	M	84	SSM	Extremities	5.55	4	No	4	$BRAF^{V600E}$	Skin (satellitoses)	SE	Yes (10)	No
**19**	F	51	NM	Trunk	3.90	4	No	3	$BRAF^{V600E}$	Visceral	SE	Yes (28)	Yes
**20**	M	60	NM	Trunk	2.30	4	Yes	4	$BRAF^{V600E}$	Nodal (regional)<br>Visceral	SE<br>Encorafenib + Binimetinib.	Yes (16)	Yes
**21**	M	61	SSM	Extremities	3.80	4	Yes	3	$BRAF^{V600E}$	Skin (in-transit)	SE	No (83)	-

**22**	F	33	NM	Trunk	1.57	3	No	1	*BRAF*^{V600E}	Skin (regional)<br>Visceral	PT	Yes (149)	Yes
**23**	F	35	SSM	Head and neck	11.15	4	Yes	10	*BRAF*^{V600E}	Visceral	RT + Verumafenib	Yes (77)	Yes
**24**	M	65	SSM	Trunk	2.60	3	Yes	5	*NRAS*^{Q61}	Nodal (regional)	SE + RT + IFN (adjuvant)	Yes (44)	Yes

SSM: superficial spreading melanoma; NM: nodular melanoma; CNS: central nervous system; SE: Surgical excision; RT: Radiotherapy; IFN: Interferon; N/A: not available; IC: Immunocriotherapy; IQ: Immunochemotherapy (Dacarbazine + gemcitabine + IL-2 + IFN); PT: Palliative treatment. *age at MMp diagnosis, **months of follow up since MMp diagnosis.

Table S2. Distribution of pathological characteristics between MMp and MMx groups.

**Cell Morphology**						**Pigmentation**						**Lymphocitic infiltrate**					
**Epithelioid**		**Spindled**		**Mixed**		**High**		**Moderated**		**Low/absent**		**High**		**Moderated**		**Low/absent**	
50		16		28		46		28		20		10		33		51	
**MMp**	**MMx**	**MMp**	**MMx**	**MMp**	**MMx**	**MMp**	**MMx**	**MMp**	**MMx**	**MMp**	**MMx**	**MMp**	**MMx**	**MMp**	**MMx**	**MMp**	**MMx**
32	18	11	5	23	5	36	10	21	7	9	11	4	6	21	12	41	10
